# Case Report: Differential Genomics and Evolution of a Meningeal Melanoma Treated With Ipilimumab and Nivolumab

**DOI:** 10.3389/fonc.2021.691017

**Published:** 2022-01-05

**Authors:** Remberto Burgos, Andrés F. Cardona, Nicolas Santoyo, Alejandro Ruiz-Patiño, Juanita Cure-Casilimas, Leonardo Rojas, Luisa Ricaurte, Álvaro Muñoz, Juan Esteban Garcia-Robledo, Camila Ordoñez, Carolina Sotelo, July Rodríguez, Zyanya Lucia Zatarain-Barrón, Diego Pineda, Oscar Arrieta

**Affiliations:** ^1^ Neurosurgery Department, Clínica del Country/Clínica Colsanitas, Bogotá, Colombia; ^2^ Foundation for Clinical and Applied Cancer Research (FICMAC), Bogotá, Colombia; ^3^ Molecular Oncology and Biology Systems Research Group (Fox-G), Universidad El Bosque, Bogotá, Colombia; ^4^ Direction of Research and Education, Luis Carlos Sarmiento Angulo Cancer Treatment and Research Center (CTIC), Bogotá, Colombia; ^5^ Clinical and Translational Oncology Group, Clínica del Country, Bogotá, Colombia; ^6^ Clinical Oncology Department, Clínica Colsanitas, Bogotá, Colombia; ^7^ Radiotherapy Department, Carlos Ardila Lulle Institute of Cancer (ICCAL), Fundación Santa Fe de Bogotá, Bogotá, Colombia; ^8^ Division of Hematology/Oncology, Mayo Clinic, Phoenix, AZ, United States; ^9^ Thoracic Oncology Unit and Personalized Oncology Laboratory, National Cancer Institute (INCan), México City, Mexico; ^10^ Radiology Department, Clinica del County/Resonancia Magnética de Colombia, Bogotá, Colombia

**Keywords:** melanoma, radiosurgery, immunotherapy, genomics, GNAQ, TERT, meningeal melanocytic tumor

## Abstract

Primary melanocytic tumors of the CNS are extremely rare conditions, encompassing different disease processes including meningeal melanoma and meningeal melanocytosis. Its incidence range between 3-5%, with approximately 0.005 cases per 100,000 people. Tumor biological behavior is commonly aggressive, with poor prognosis and very low survivability, and a high recurrence rate, even after disease remission with multimodal treatments. Specific genetic alterations involving gene transcription, alternative splicing, RNA translation, and cell proliferation are usually seen, affecting genes like BRAF, TERT, GNAQ, SF3B1, and EIF1AX. Here we present an interesting case of a 59-year-old male presenting with neurologic symptoms and a further confirmed diagnosis of primary meningeal melanoma. Multiple therapy lines were used, including radiosurgery, immunotherapy, and chemotherapy. The patient developed two relapses and an evolving genetic makeup that confirmed the disease’s clonal origin. We also provide a review of the literature on the genetic basis of primary melanocytic tumors of the CNS.

## Introduction

Melanocytic tumors that originate in the meninges are rare. These tumors might present as focal (melanomas) or diffuse conditions (melanocytosis). Staging might vary from low grade to high grade malignant stages. These tumors are frequently diagnosed in patients >40 years old and in females. The most common places where it develops include the cervical and thoracic spine and the posterior fossa (1–4). When its location is the leptomeninges, clinical symptoms and signs are non-specific (5), the gold standard for diagnosis is the evidence of tumor cells present in cerebrospinal fluid, with a sensitivity of CSF cytology of 50% in the first puncture and 98% in repeated punctures (5, 6). Magnetic resonance imaging (MRI) has a sensitivity and specificity of 77%, and a typical leptomeningeal contrast enhancement might be the most frequent finding (5).

Primary meningeal melanocytic neoplasms (PMMs) share genetic and molecular characteristics with uveal melanomas (UMs) (1, 3), which have been found to recur frequently and to have an aggressive behavior with leptomeningeal spread (2). PMMs share mutations with UM. Some of the genes affected include GNAQ/GNA22 or GNA11 (7, 8) (paralogue genes), and to a lesser degree, SF3B1 and EIF1AX (2, 3), while BRAF mutations and TERT promoter mutations are rare (9).

Common mutations have been reported in primary meningeal melanomas with NRAS, SFEB1, and EIF1AX and coexisting with GNAQ or GNA11 (3). Also, the loss of chromosome 3 and BAP1 mutations have been detected in some melanocytic meningeal tumors (2, 3). Therefore, these molecular differences have implications in targeted therapy (3). Meningeal melanomas have specific methylation that allows them to be discriminated from other tumors of the central nervous system. However, there is still no information to establish an adequate primary meningeal tumor profile (2). Understanding the genomic alterations of meningeal melanomas can help improve the diagnosis and treatment.

Among therapies for PMMs, focal radiotherapy, intrathecal and systemic chemotherapy have been proposed, with just a partial benefit. A study assessing radiotherapy’s effectiveness combined with checkpoint inhibition using ipilimumab for leptomeningeal melanoma metastases (LMM) showed that complete responses could be achieved (5). LMM and PMMs usually have a poor prognosis with a median overall survival of 2-4 months. Nevertheless, the use of multimodal interventions (surgery, radiotherapy/radiosurgery, targeted therapy, and immunotherapy) has shown a global benefit in managing this type of tumor (10). Here, we present the case of a patient with a PMM considering its clonal evolution throughout various surgical interventions, radiosurgery, the use of adjuvant ipilimumab, the combination of ipilimumab/nivolumab and temozolomide. We also discuss the current evidence on the genomics of PMMs and their treatment and contrast it with the findings taken from our patient. These data provide an insight into new and alternative ways to treat PMMs.

## Case Presentation

**Figure 1:**
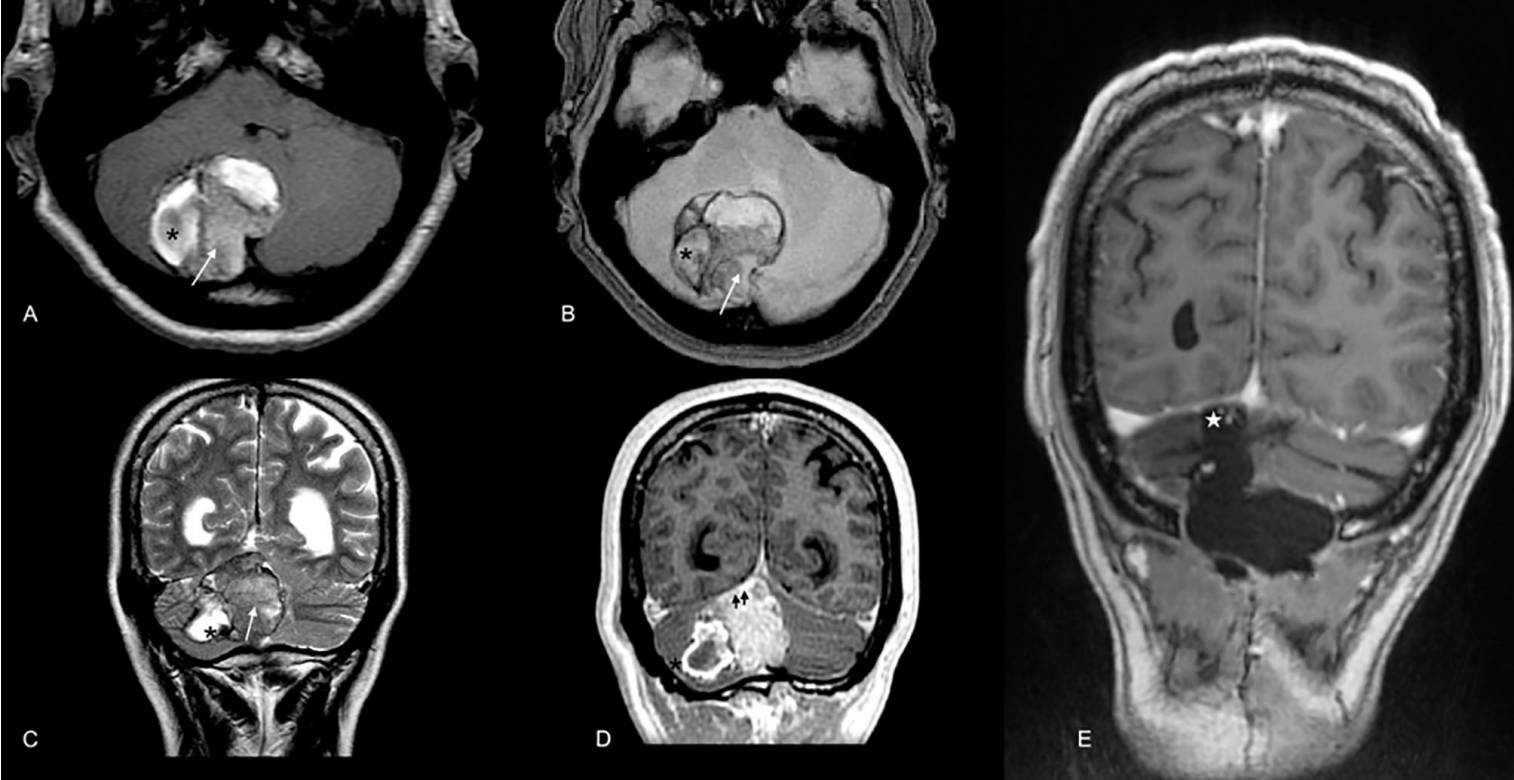
Brain MRI scan at diagnosis in 2016. Extra-axial bulky solid mass with a hypercellular solid component in the right paravermian location depicted by the arrow in **(A)** (axial T1WI), **(B)** (GRE), and C (T2WI). Note the hypointense components in T2WI **(C)** and blooming effect in GRE **(B)** highly suspicious for melanic or pigmented components. Also, note the coexistent intraparenchymal hematoma [* in **(A–D)**]. Mass was firmly attached to dural surface compressing right transverse dural sinus (doble arrowhead in D, postcontrast T1WI). In the post-operative scan [**(E)**, T1 WI postcontrast], no macroscopic evidence of residual disease in the surgical cavity was noted [white star in **(E)**].

**Figure 2:**
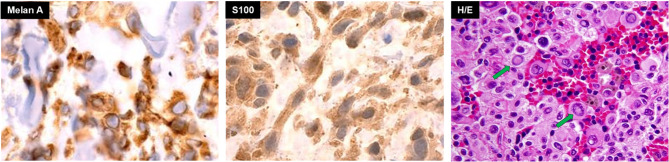
Basal pathology of meningeal hyperpigmented lesion from the posterior fossa compatible with a primary melanoma of the meninges. Green arrows in H/E image show large nucleoli in cells with a high nucleus-cytoplasm ratio.

In 2016, a 59-year-old male presented with acute severe headaches, nausea, vomiting, gait instability, and functional limitation. An MRI brain scan showed a bulky solid mass in an extra-axial location, firmly adhered to the right transverse sinus. The solid portion (measuring 38x22x19 mm) coexisted with an intraparenchymal hematoma. The mass effect of the complex partially collapsed the fourth ventricle causing ascending transtentorial herniation. Additionally, cerebellar tonsil herniation through the foramen magnum was noted (**Figure 1**). The tumor was completely resected, and histology was consistent with a meningeal melanocytic tumor of high-grade malignancy. Histologically, the tumor was moderately pigmented with a lobular architecture associated with prominent vascularization and interconnecting vascular lakes showing melanoma features. A high nuclear-cytoplasmic ratio and prominent nucleoli were observed as well as multinucleation. There were five mitoses per 10 HPF. Areas of hemorrhage and apoptosis were identified, but there was no necrosis. The tumor cells expressed S100, HMB45, and Melan A (**Figure 2**). Ki-67 proliferative index was 15%, and nuclear BAP1 immunohistochemistry expression was utterly negative in melanocytes, with positive staining of endothelial cells (images not available). The clinical, mucosal, and ophthalmological examination did not reveal other melanoma localizations.

**Figure 3:**
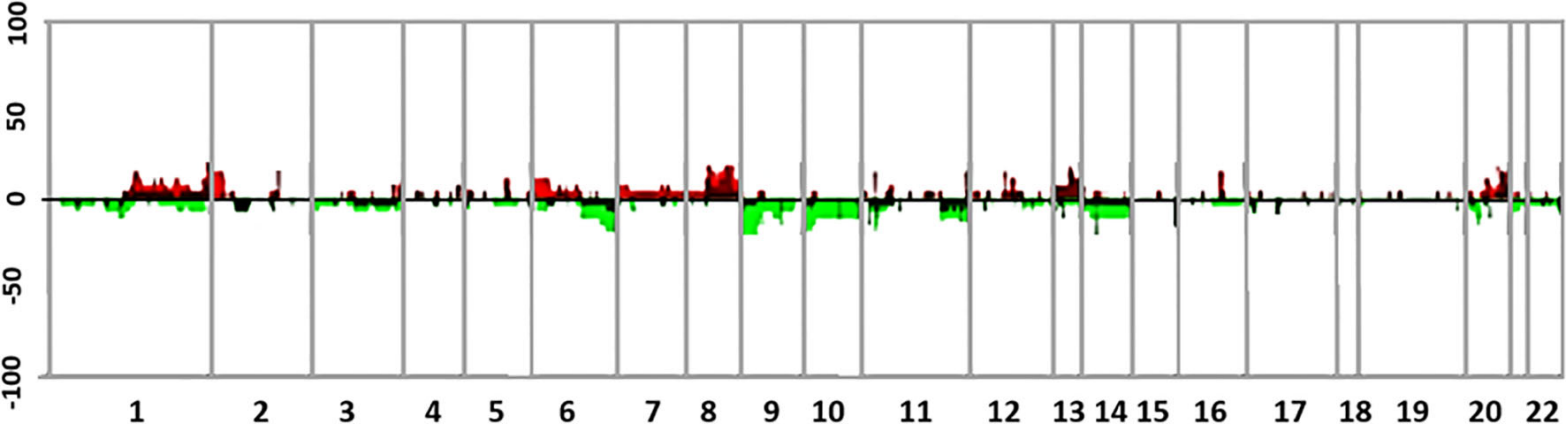
Summary of genomic profiles of four different samples from the case. Samples correspond to the meningeal melanoma at diagnosis and its three further recurrences with their chromosomal alterations. The recurrence of CNAs across the samples in segmented data (y-axis) is plotted for each probe (4) evenly aligned along the x-axis in chromosomal order. The percentage of samples harboring gains, amplifications, losses, and deletions for each locus is depicted according to the following scheme: dark red (gains with a log_2_ ratio > = 0.15) and green (loss with a log_2_ ratio < = −0.15) and are plotted along with bright red (amplifications with a log_2_ ratio ≥ 0.4) and bright green (deletions with log_2_ ratio ≤−0.4).

**Figure 3** shows the comparative genomic hybridization array (aCGH) analysis of the four samples obtained from surgical resections, including monosomy of chromosome 3 and X, a total gain of chromosome 20, a high-level gain of chromosome 8q, and segmental losses on chromosomes 1p and 6q. The primary melanocytic tumor harbored a somatic mutation in GNAQ (c.626A>T), alteration present in 0.07% of AACR GENIE cases (https://portal.gdc.cancer.gov/), of uveal melanoma, ocular melanoma, cutaneous melanoma, and central nervous system melanoma. Besides, a commutation was found in the eukaryotic translation initiation factor 1A (EIF1A), a gene that encodes a protein that acts as an essential eukaryotic translation initiation factor. The G15D mutation (c.44G> A) is found in up to more than 30% of UMs and in primary melanomas of the meninges, where it usually co-occurs with other mutations in SF3B1, as in the present case (R625H mutation, c.1874G> A) (**Supplementary Data** includes a detailed description of the methods performed for the NGS assessment on the Ion Torrent™ Oncomine™ Comprehensive Assay Plus). As previously reported, the primary tumor’s genomic analysis did not reveal mutations in BAP1, BRAF, NRAS, HRAS, KIT, and TERT. Neither microsatellite instability was evidenced, and Tumor Mutation Burden (TMB) was estimated at 3 Mut/Mb.

Postoperatively, patient underwent radiosurgery (gamma knife 16 Gy) on the cerebellar cavity, without complications, followed by four cycles of intravenous ipilimumab 10 mg/kg administered every three weeks for four doses, and then every 12 weeks until one year of treatment. After four cycles of ipilimumab, he developed moderate ipilimumab-induced hepatitis successfully treated with high dose PO corticosteroids (prednisone 1 mg/kg/day for ten days) subsequent tapering.

**Figure 4:**
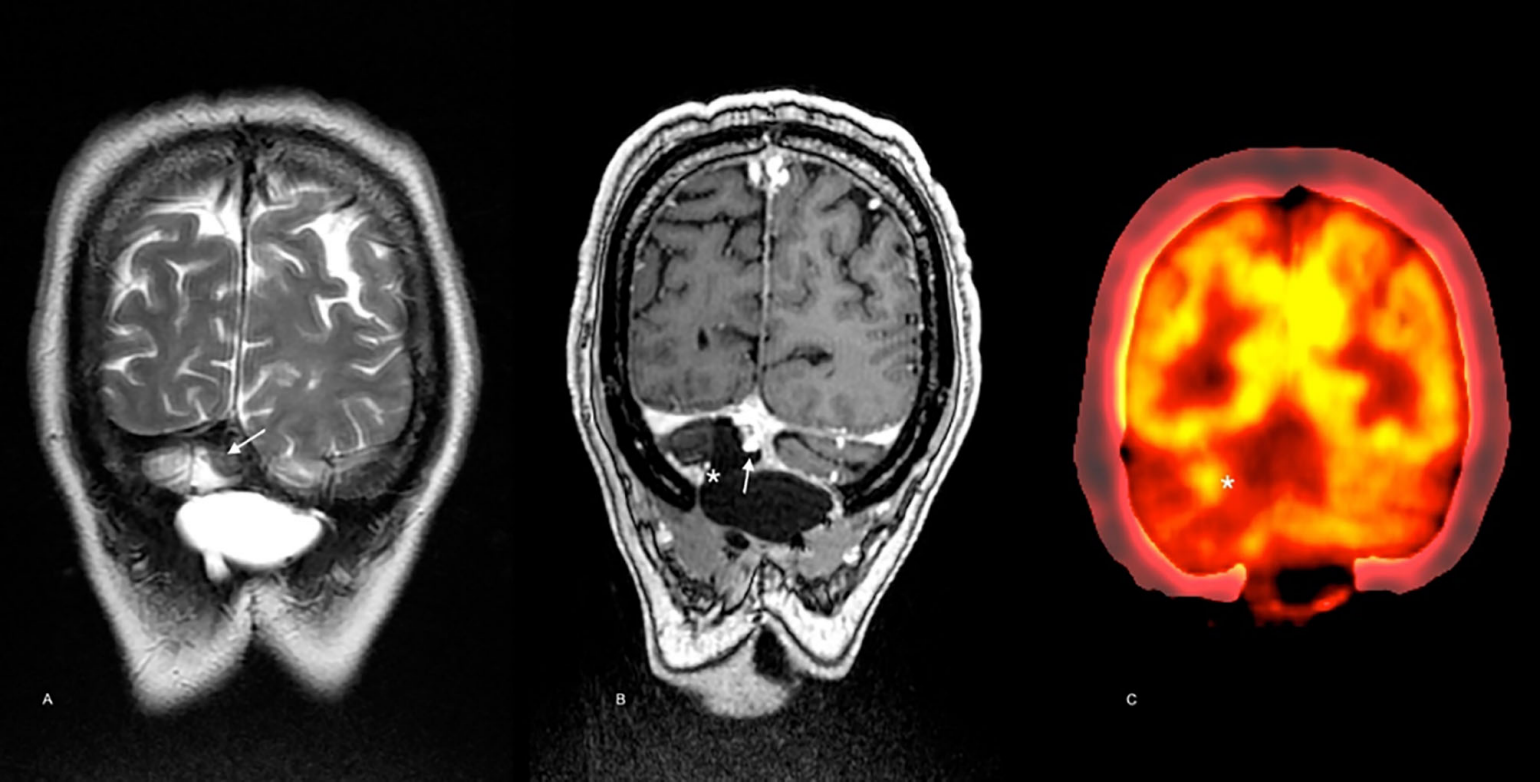
Imaging follow-up 23 months after diagnosis. Brain MRI scan showed a solid nodule below the torcular Herophili at the midline [arrow in **(A)**, T2WI, and **(B)**, postcontrast T1WI]. Note the low signal in the nodule in A (T2WI) and the avid contrast enhancement in B (postcontrast T1WI) in a similar pattern as in the pre-operative scan. In the medial margin of the surgical cavity [* in **(C)**] contrast enhancement may be noted matching the hypermetabolic focus seen on PET-CT [* in **(D)**] consistent with local recurrence.

**Figure 5:**
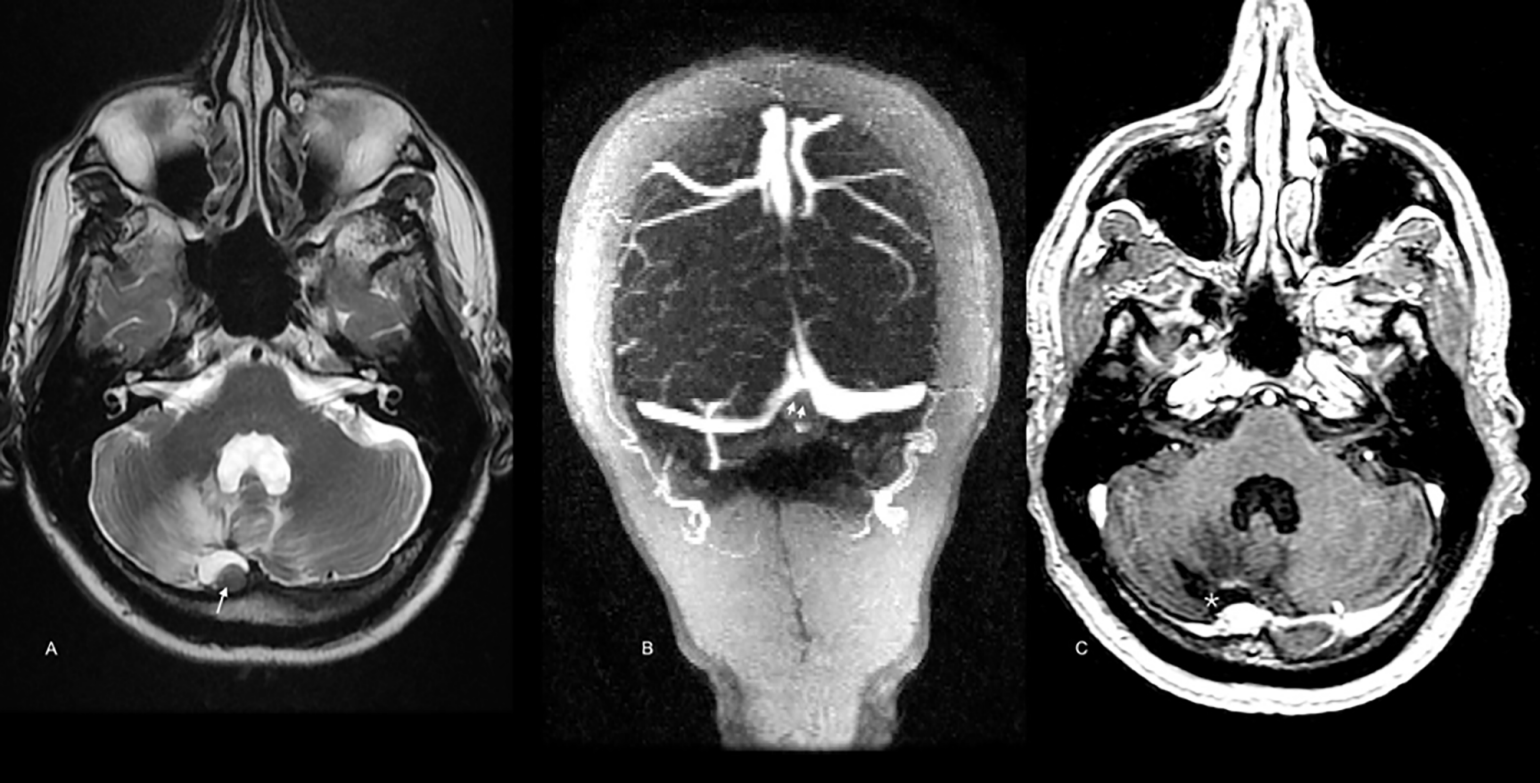
Brain MRI scan after secondary resection. A similar low signal nodule in the surgical cavity is depicted [arrow in **(A)**, T2WI]. According to new onset of cephalalgia, the torcula’s extrinsic compression was demonstrated [double arrowhead in **(B)**, venous-phase angio-MRI]. In **(C)** (postcontrast T1WI), meningeal and surgical cavity enhancement is also noted (star).

After 23 months, the patient presented with recurrent symptoms. Follow-up imaging showed vasogenic edema involving the right cerebellar hemisphere and the vermis, with a small marginal nodule visible on MRI and PET/CT (SUVmax 3.2) (**Figure 4**). In March 2018, he was taken to an optimal secondary surgery that confirmed recurrent melanoma with an expression of Melan A, HMB45, S100, SOX10, and MITF. Ki-67 was quantified at 30%. The second sample’s genetic analysis revealed five new alterations in the TERT promoter (C228T), SDE, PDRX2, CHIT1, and TNIP genes. As a complication, he had a CSF cyst plus bacterial meningitis that delayed the execution of new radiosurgery with gamma knife (16 Gy). Then, he was started on nivolumab, achieving a progression-free survival (PFS) of 7.2 months. He developed meningeal progression with new nodules and meningeal thickening attached to the torcula causing partial compression of the right transverse sinus (**Figures 5A, B**). A cfDNA analysis was carried out by NGS in the cerebrospinal fluid, finding only the TERT promoter’s mutation. In parallel, he started with ipilimumab (1 mg/kg) plus Nivolumab (3 mg/kg) every three weeks following data from the CheckMate 511 phase IIIb/IV trial. Initially, no limiting toxicity was found; however, he had a recurrence of grade 3 hepatitis just after the fourth cycle when steroids were administered again. Subsequently, he received 15 cycles of Nivolumab 480 mg every 28 days until February 2020. An irregular nodular enhancement focus was then found in the right paravermian cerebellar postsurgical cavity, related to local tumor relapse with perilesional vasogenic edema (**Figure 5C**). He was taken to a third neurosurgical intervention without complications but with greater neurological involvement concerning slight ataxia and dysmetria.

**Figure 6:**
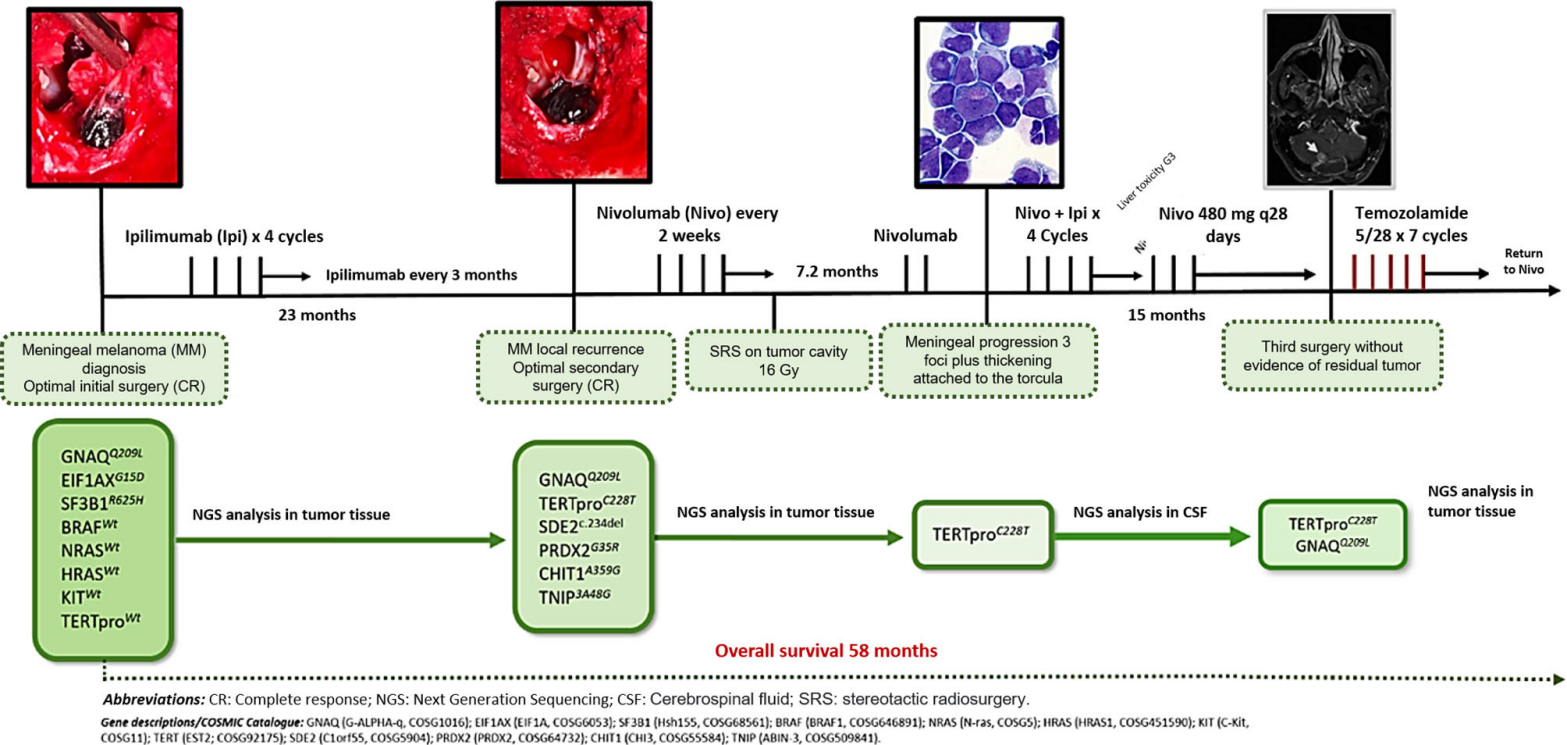
Graphic evolution of the clinical case and of the tumor genomics evaluated by NGS in tumor tissue and CSF.

After finding persistence of the GNAQ and TERT alterations in tumor tissue, he began treatment with temozolomide on the 5/28 schedule, maintaining the response to date. At that time, he completed 58 months of survival. **Figure 6** describes the timeline from diagnosis to the present, including the different genomic findings. Decision of initial and subsequent therapies were made based on progression development. No targeted therapies were used.

## Discussion

In this case, a patient with non-specific neurological symptoms is presented. Due to their persistence, an MRI is performed where a heterogeneous lesion of extra-axial location and adhere to the transverse sinus with cerebellar involvement is evident; surgical intervention was done early, showing a malignant melanoma macroscopically. Immunohistochemical findings showed the expression of Melan-A, HMB, S100, and MITF and negativity for AE1/AE3. At diagnosis, the presence of BRAF, NRAS, HRAS and KIT mutations was ruled out, and during disease evolution, several alterations were found in GNAQ, EIF1AX, SF3B1, SDE2, PRDX2, CHIT1, TNIP, and TERT. Treatment included radiosurgery and checkpoint inhibition with ipilimumab and nivolumab with an adequate response.

The development of extracutaneous primary melanomas results from malignant transformation of neural crest cells that might have scattered throughout mucous membranes, eyes, and the leptomeninges during cell migration in embryogenesis (4, 11). PMM is a rare type of tumor. Its presentation is associated with focal/spread meningeal lesions or metastases. Evidence regarding the biology and clinical features of this condition is scarce; however, specific analyses have shown that these types of tumors present with a particular genetic makeup that allows for an adequate and comprehensive therapeutic approach. The first case of PMM was described by Virchow in 1859, with very few cases reported later. PMMs are usually seen in adults, with a higher prevalence among those over the fourth decade of life. Pediatric patients are extremely rare, accounting for approximately 0.1% of all pediatric central nervous system tumors (4).

These tumors have an uncertain biological behavior; most of them are aggressive, although they depend on their genetic background (12). These tumors’ behavior is not well defined either; however, prognostic markers such as BAP1 mutations and chromosome loss have been proposed (2, 9). Copy number variations such as 6p gain, 6q loss, chromosome 8 gain, 1p loss, and 1q gain have been reported in some meningeal melanocytic tumors. This becomes important given that they have predictive value, as the gain of chromosome 8 and loss of 1p is associated with a worse prognosis (9).

A cerebrospinal fluid sample, in some cases, may be the first and only sample necessary to make the diagnosis of meningeal melanoma, as different diagnostic biomarkers like Melan A, HMB-45, and MITF can be found in this fluid (13). Mutations in GNAQ and GNA11 are commonly found in adults with PMM, while children usually present with NRAS^Q61K^ (4).

**Figure 7:**
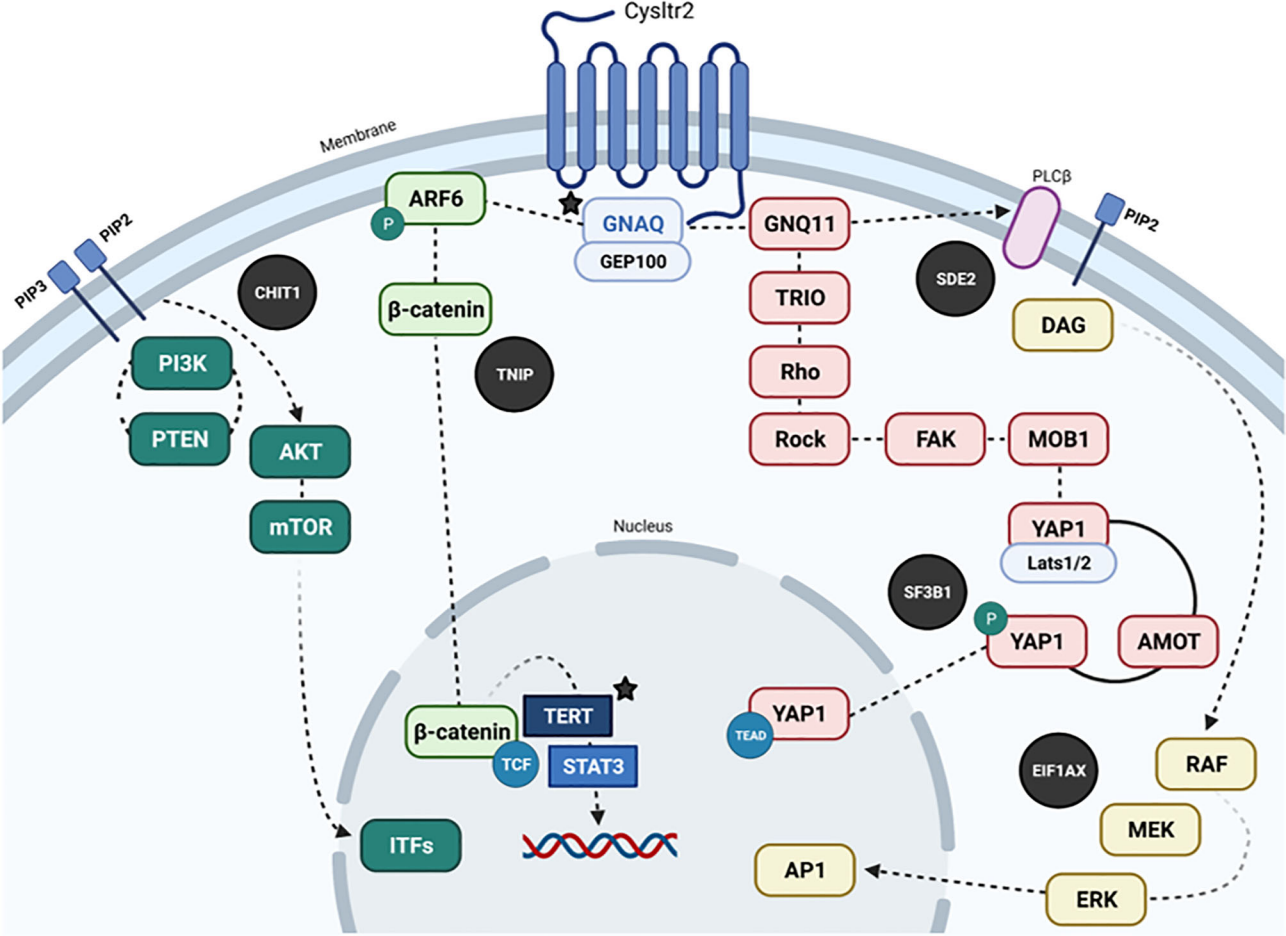
Dysregulated pathways in PMMs (in black circles, other mutated genes are highlighted with their protein representation, and colocalization parallel to the main signaling pathways). Recurrent mutations in GNAQ, PLCβ4, and CYSLTR2 are mutually exclusive and trigger Gαq signaling and related pathways (Akt/mTOR, Wnt/β-catenin, Yes-associated protein (YAP), and MAPK pathways). In brief, GNAQ mediates signals between the G protein-coupled receptor (GPCR) and downstream effectors. Receptor activation by ligand binding causes the activation of GNAQ by catalyzing the release of GDP and binding of GTP. In its active form, GTP-bound GNAQ causes the release of the beta and gamma subunits of the heterotrimeric G-protein. GTP-GNAQ and beta and gamma subunits transfer the receptor-mediated signal to downstream effectors through secondary messengers, which participate in diverse signaling pathways to evoke different effectors. The known effectors for GNAQ include PLC beta, p63-RhoGEF, Trio, and Duet. GNAQ has been shown to activate the MAP kinase pathway, possibly *via* DAG-mediated activation of protein kinase C isoforms. GNAQ has an intrinsic GTPase domain at the C terminus, which causes the hydrolysis of GTP to GDP, and the G-alpha-GDP re-associates with G-beta and G-gamma subunits. Somatic mutations in GNAQ have been described in uveal and meningeal melanocytic neoplasias. In uveal melanoma, 97% of the hotspot mutations cause the amino acid substitution Q209L (data similar to rare cases originating in the meninges); the other 3% of mutations generate amino acid change R183Q. The Glutamine 209 of GNAQ is similar to residue 61 of RAS protein. The Q209 and R183 mutations cause a complete or partial loss of intrinsic GTPase activity, thereby locking the protein in a constitutively active form. Q209 and R183 mutations occur in a mutually exclusive pattern in human neoplasia. Mutations in GNAQ are also mutually exclusive from the hotspot mutations in GNA11, which belongs to the same family and shares 90% sequence homology. GNAQ mutations are not concurrent with other common oncogenic mutations in BRAF, NRAS, or KIT found in common melanomas. CYSLTR2, Cysteinyl leukotriene receptor 2; PIP3, Phosphatidylinositol, 3,4,5)-trisphosphate; PIP2, Phosphatidylinositol 4,5-bisphosphate; GNAQ, G protein subunit alpha q; ARF6, ADP-ribosylation factor 6; GNQ11, G protein subunit alpha 11; GEP100, ADP-Ribosylation Factor - Guanine nucleotide-Exchange Protein; PI3K, Phosphatidylinositol 3-kinase; PTEN, phosphatase and tensin homolog; AKT, AKT serine/threonine kinase; mTOR, mechanistic target of rapamycin kinase; CHIT1, Chitinase 1; TNIP, TNFAIP3 interacting protein; TRIO, Triple functional domain protein; Rho, Rho factor; Rock, Rho kinase; FAK, PTK2 protein; MOB1, MOB kinase 1A; YAP, Yes-associated protein 1; AMOT, Angiomotin; TERT, Telomerase reverse transcriptase; RAF, RAF kinase; MEK, Mitogen-activated protein kinase; ERK, extracellular signal-regulated kinase; STAT3, Signal transducer and activator of transcription 3; SF3B1, Splicing factor 3B subunit 1; EIF1AX, Eukaryotic translation initiation factor 1A.

This NRAS mutation is most probably developed during embryogenesis, throughout the post-zygotic stage of neural crest cells, before migration to the skin and leptomeninges, which could condition NRAS mosaicism (4). In a case series, Küsters-Vandevelde et al. showed NRAS mutations in a patient with a melanocytic CNS tumor and congenital melanocytic nevus, with an SF3B1 mutation only in the CNS tumor but not in the melanocytic nevus. On the other hand, some cases of melanocytomas with meningeal seeding plus SF3B1 mutations and associated GNAQ were reported (3). A point mutation in EIF1AX was also reported in five primary meningeal melanomas (3). Consistently, EIF1AX mutations were mutually exclusive with SF3B1 mutations but coexisted with GNAQ or GNA11 alterations suggesting that they occur in the late phase of tumorigenesis (3, 14). SF3B1 gene mutations in PMMs occur mainly at codons 625 or 634. They are regularly associated with a disomy for chromosome 3, accompanied by GNAQ or GNA11 mutations and overexpression, and to a lesser extent with NRAS mutations (3). The GNAQ, GNA11, and NRAS mutations are believed to play a critical role in the initiation of PMMs tumorigenesis, while the SF3B1 conversion seems to be a later event (3). Curiously, in our case, the baseline coexistence of the GNAQ^Q209L^, EIF1AX^G15D^, and SF3B1^R625H^ mutations was found, a profile that had not been previously described in PMMs, or their uveal counterparts. **Figure 7** integrates GNAQ-related signaling pathways in PMMs. Griewank et al. analyzed a large set of CNS melanocytic tumors using techniques like mutation analysis, copy number alterations and DNA methylation profiling. They included PMMs, UMs, CNS cutaneous melanoma metastases and blue nevus-like melanomas. They found that EIF1AX, SF3B1, and BAP1 mutations in UM are associated with favorable, intermediate, and poor prognosis, respectively. They also showed that EIF1AX, SF3B1, and BAP1 mutations in PMMs don’t seem to match accordingly with an expected histologic pattern, this might explain why in our patient, there were no BAP1 mutations but a negative expression in IHC. The researchers also demonstrated that PMMs harboring chromosome 3 loss and BAP1 alterations (mutations or IHC loss), should be considered as high risk, with a high malignant potential. This is in fact the situation of our patient. Also, when there are no alterations in EIF1AX, SF3B1, and BAP1, or there are only mutations in EIF1AX with wild-type versions of SF3B1 and BAP1, the prognosis is favorable (15).

Additionally, in our case, a mutation of the somatic telomerase reverse transcriptase gene (TERT) was identified. This mutation has been evidenced in approximately 80% of cases of patients with sporadic and familial melanoma (12, 16); although this mutation is rare in PMMs, it is very pervasive, and it is believed to increase gene expression, generating a positive selection of malignant cells. Besides, a joint expression has been shown with BRAF and NRAS, which activate melanoma oncogenesis, and TERT activation is believed to enhance melanocyte immortalization (12, 16, 17). TERT promoter mutations are found more frequently in sun-exposed sites and show mutations that could be consistent with UV-induced cytidine-to-thymidine transitions (6), it has been suggested that these mutations might occur early in the development of cutaneous melanoma. As the primary melanoma occurred on sun-exposed skin, it is somewhat surprising that a UV-induced TERT promoter was not detected in the primary lesion. However, as C228T mutations are also frequently found in UV-protected internal malignancies, it is possible that the melanoma acquired its TERT promoter mutation after metastasizing (6, 18). Alternatively, the C228T mutant cells could have been present as a small subset of the primary lesion, which was not detected by initial gene sequencing, but then became the dominant cell type by the time the melanoma was progressive.

Leptomeningeal melanoma remains a devastating complication, and its control is a tremendous unmet clinical need since progression results in rapid neurological decline and death. The diagnosis can be difficult and is often made on radiological findings without confirmation of CSF cytology. Arasaratnam et al. recently reported the outcomes of fourteen patients with extracranial melanomas and meningeal involvement (10). Almost all had BRAF mutations (79%). The median time from diagnosis of metastatic melanoma to confirmed leptomeninges’ involvement was 5.7 months, and all but one patient received local therapy, systemic therapy, or both. The median overall survival (OS) from diagnosis of meningeal disease was 5.2 months, and 12-month OS was 21%. Additionally, immunotherapy was administered to 64% of patients (two ipilimumab, five anti-PD1 antibodies, and one both) with a median OS for those who received ipilimumab of 3.0 months. The patients that received anti-PD-1 antibodies appeared to live longer than those that did not (median OS 7.1 months vs. 2.9 months) (10). Central nervous system involvement in patients with UMs is extremely rare and only five cases has been described in the literature to date (19). Patients with UM leptomeningeal disease typically have a median OS of ~10 weeks and derive benefit from intrathecal interleukin-2 (IT IL-2), whole brain radiation and ipilimumab (20).

Given the biological similarity between UMs and PMMs, the therapeutic results on the former might be extrapolated. Previously, Heppt et al. identified and analyzed seven expanded access programs (EAPs) (n=162), 4 phase II trials (n=171), and 1 phase Ib trial of immunotherapeutic interventions in UM patients (21). Ipilimumab monotherapy was assessed at 3 mg/kg in 5 trials (n=186) with a 0 to 5% response rate. Besides, two reports investigated ipilimumab at 10 mg/kg (n=45) with radiological responses observed in 0 to 6.5%. The median progression-free survival (PFS) was below 3 months in both groups, and the median OS was 5.2-9.8 months. Similarly, two studies investigated pembrolizumab or nivolumab with overall response rates (ORRs) of 30% and 6%, respectively. Data on combined ipilimumab and PD-1 inhibition were available from one EAP, but no response was observed with a median PFS of 2.9 months (21). Even though UMs and PMMs share a wide array of driver mutations, treatment outcomes might vary, this is mainly due to the physiological immune privilege of the eye (22).

The systematic characterization of the immune profile of UMs made it possible to find that tumors with the greatest potential for response to immunotherapy were those that showed a higher level of CD3+, CD8+, FoxP3- T cells, and CD68+ macrophages. Also, the analysis of RNAseq expression profiles by NanoString revealed significant differences in a set of immune markers between responders, including a group of genes relevant to the interferon-γ signature, particularly, the suppressor of cytokine signaling-1 (identified as a marker potentially contributing to the response to immunotherapy (23). Paradoxically, recurrence-free survival (RFS) in patients with UMs seems to be related to higher PD-L1 expression and fewer tumor-infiltrating lymphocytes (TILs). However, the cellular response to PD-1 inhibitors and disease control in UMs does appear to be dependent on IFN-γ levels (24). This information has not been extrapolated to PMMs, but it could be used in the future as a factor to stratify the population of patients who are candidates for combination immunotherapy. In our case, the patient had adequate control of the disease after the use of adjuvant ipilimumab (10 mg/kg), and with ipilimumab/nivolumab after recurrence. Other recent reports have demonstrated the value of immunotherapy in patients with primary leptomeningeal melanomatosis and melanomas (25).

Notably, other strategies were developed, considering that the typical mutations in GNAQ/GNA11 in UMs and PMMs lead to constitutive activation of the MAPK and PI3K/AKT pathways (26). Thus, logical approaches considered downstream targeted therapies against effector proteins, such as MEK and AKT. Some clinical trials were developed based on this rationale of inhibition of downstream Gαq (**Supplementary Table 1**). In this context, selumetinib (an oral selective MEK1/2 inhibitor) was tested against chemotherapy (temozolomide or dacarbazine) in a phase 2 trial, and in combination with dacarbazine in the phase 3, multicenter, and randomized SUMIT trial. Unfortunately, both studies showed limited clinical activity (ORR 14% and 3%, respectively) in advanced UM patients (27, 28). Subsequently, the MEK inhibition trametinib was tested alone or in combination with the AKT inhibitor GSK2141795 in a phase 2 trial, including patients with advanced UM (29). The combination did not improve the clinical outcomes since patients in the trametinib arm (*n* = 18) achieved an ORR of 5.5% compared to 4.8% in the combined arm (*n* = 21). The median PFS was 3.6 months in both groups. Based on the concept that UMs (and probably PMMs) normally synthesize and secrete vascular endothelial growth factor (VEGF), an additional targeted therapy tested was the oral multi-kinase inhibitor sunitinib (30). Scheulen et al. developed a phase 2 trial recruiting 118 chemonaive patients with metastatic UM. Unfortunately, only two cases had a partial response (1.7%), 78 had a stable disease (66.1%), and the median PFS was 5.5 months (31).

## Conclusions

In conclusion, although PMM is a rare entity and its presentation is aggressive in most cases, understanding gene expression, signaling pathways, and determining cancer genomics enables better performance in targeted treatments; however, more studies are still required better to understand the pathogenesis and early treatment of this pathology. Furthermore, the comprehensive and integrated approach with neurosurgery, radiotherapy, pathology, clinical oncology, and the early initiation of medications is fundamental to improve survival, prognosis and decrease disease recurrences (32).

## Data Availability Statement

The original contributions presented in the study are included in the article/**Supplementary Material**. Further inquiries can be directed to the corresponding author.

## Ethics Statement

Written informed consent was obtained from the individual(s) for the publication of any potentially identifiable images or data included in this article.

## Author Contributions

RB, AC, and OA were in charge of ideation, analysis, writing and correction. NS, AR-P, JC-C, LeR, LuR, AM, JEG-R, CO, CS, JR, ZZ-B, and DP also participated in analysis, writing and correction. All authors approved the final version of the manuscript before submission.

## Conflict of Interest

The authors declare that the research was conducted in the absence of any commercial or financial relationships that could be construed as a potential conflict of interest.

## Publisher’s Note

All claims expressed in this article are solely those of the authors and do not necessarily represent those of their affiliated organizations, or those of the publisher, the editors and the reviewers. Any product that may be evaluated in this article, or claim that may be made by its manufacturer, is not guaranteed or endorsed by the publisher.
