# Supplementary material for: Case Report: Differential Genomics and Evolution of a Meningeal Melanoma Treated With Ipilimumab and Nivolumab
Source: Front Oncol. 2022 Jan 5;11:691017. doi: 10.3389/fonc.2021.691017 (PMC8766339; doi:10.3389/fonc.2021.691017)
Supplement: Supplementary file 1 [file DataSheet_1.docx]

**Supplementary data**

**Histological analysis**

Immunocytochemical analysis was performed using standard protocols. Briefly, 5-µm sections were cut, placed on electrostatically charged glass slides, and deparaffinized. Staining for the remaining primary antibodies was performed using the Ventana ES automated immunohistochemistry system and the Ventana DAB Detection Kit (Ventana Medical Systems, Tucson, AZ). The primary antibody used included antibodies to S-100 protein (clone S-100, Ventana Medical Systems), HMB-45 antigen (clone gp100, Ventana Medical Systems), tyrosinase (clone T311, Neomarkers, Fremont, CA), and Melan-A (clone A013, Neomarkers). All 4 antibodies were prediluted and incubated with the tissue section for 1 hour at room temperature. Counterstaining was performed with hematoxylin. Appropriate positive and negative controls were used; the latter was achieved by omitting the primary antibodies.

**Sample collection of cerebrospinal fluid (CSF)**

**CSF cytology examination**

About 0.5 ml CSF was centrifuged at 750 r/min for 4 min (Therm-4, Shandon Cytospin, US). After naturally drying on the slide, the deposit was dyed with May–Grünwald–Giemsa liquid (MGG) and Alcian blue staining, according to previously published protocols, and observed under a light microscope (OLYMPUS-BX41). The determination of the positive result was considering cells that had an irregular size and shape, and contained large and polymorphic nuclei with a lobulated state and malformed buds. The chromatin size increased with the basophilic coarse particles, and the mitotic activity was enhanced with aberrant mitosis. The nuclear membrane was usually thickened with a saw-tooth-shaped and wear edge.

**CSF samples and processing**

The CSF samples were collected from the patient and placed into EDTA tubes (5 ml), according to our routine protocols. Then, these were centrifuged for five minutes at 1000 g. The pellet was stored at −20 °C, while the supernatant was centrifuged at 10,000 g for an additional 30 min, according to a previous study (1). The supernatant was transferred into pre-labeled cryotubes and stored at −80 °C. Next, the ctDNA was extracted from at least 5 mL of the CSF supernatant using a QIAamp Circulating Nucleic Acid kit (QIAGEN), according kit instructions, and the ctDNA was quantified using a Qubit2.1 Fluorometer and Qubit dsDNA HS Assay kit (Life Technologies, Carlsbad, CA, USA).

**Ion Torrent**™ **Oncomine**™ **Comprehensive Assay**

**Nucleic acid extraction and quantification**

DNA was extracted from all samples obtained from the meningeal melanoma at different times in the evolution of the patient's disease, using QIAmp DNA FFPE Tissue Kit (Qiagen) following manufacturer's protocol (excluding deparaffinization). Dual DNA and RNA isolation was performed from tumor tissues using the RecoverAll Total Nucleic Acid Isolation Kit for FFPE (Thermo Fisher Scientific). DNA and RNA concentrations were determined by fluorometric quantitation using Qubit 2.0 Fluorimeter with Qubit DNA dsDNA BR Assay Kit and Qubit RNA BR Assay Kit (Qiagen) as appropriate.

**Next-generation sequencing**

Complementary DNA (cDNA) synthesis before library preparation for RNA panel was carried out using SuperScript™ VILO™ cDNA Synthesis Kit (Thermo Fisher Scientific, 11754050). Library preparation was carried out using the Oncomine Assay™ (comprising the DNA Oncomine™ Assay (Thermo Fisher Scientific) and RNA Oncomine™ Fusions assay (Thermo Fisher Scientific)) following manufacturer’s instructions using a total of 10 ng input DNA and or RNA per sample (minimum 0.83 ng/μl sample DNA concentration). A maximum of four representative DNA samples were prepared per run on an Ion 318™ v2 chip (Thermo Fisher Scientific, catalog no. 4488150). The DNA panel can identify hotspot mutations in over 500 genes across all classes of somatic variants. The RNA panel can identify rearrangements and copy number variants (CNVs). Template preparation was performed on the Ion Chef System (Thermo Fisher Scientific) using the Ion PGM Hi-Q Chef Kit and/or the Ion One Touch™ 2 System using the Ion PGM Template OT2 200 Kit. Sequencing was performed using the Ion PGM Hi-Q Sequencing Kit on the Ion Torrent Personal Genome Machine (Ion PGM).

**Data analysis**

Analysis was carried out using Ion Torrent Suite^™^ Browser version 5.0 and Ion Reporter^™^ version 5.0. The Torrent Suite^™^ Browser was used to perform initial quality control including chip loading density, median read length and number of mapped reads. The Coverage Analysis plugin was applied to all data and used to assess amplicon coverage for regions of interest. Variants were identified by Ion Reporter filter chain 5% Oncomine^™^ Variants (5.0)*.* A cut off of 500X coverage was applied to all analyses. All identified variants were checked for correct nomenclature using Alamut Visual v.2.7.1 (Interactive Biosoftware). Any discrepancies in variant identification, between Ion Reporter and Alamut, were validated manually using the Integrative Genomics Viewer and NextGENe® v2.4.2 (SoftGenetics®) (2,3). For the purposes of this validation, amplicons covering clinically actionable regions with known mutation status (termed target amplicons) were assessed as a subset of all amplicons (amplicons which target hot spot variants, i.e. SNVs and indels) covered in the Oncomine^™^ Focus hot spot BED file.

**References**

1. Pentsova EI, Shah RH, Tang J, et al. Evaluating Cancer of the Central Nervous System Through Next-Generation Sequencing of Cerebrospinal Fluid. J Clin Oncol. 2016 Jul 10;34(20):2404-15. doi: 10.1200/JCO.2016.66.6487. Epub 2016 May 9. Erratum in: J Clin Oncol. 2017 Jun 10;35(17):1972.
2. Thorvaldsdóttir H, Robinson JT, Mesirov JP. Integrative genomics viewer (IGV): high-performance genomics data visualization and exploration. *Brief Bioinform.*2013;14(2):178–192. doi: 10.1093/bib/bbs017.
3. Robinson JT, Thorvaldsdottir H, Winckler W, Guttman M, Lander ES, Getz G, et al. Integrative genomics viewer. *Nat BiotechNOL.*2011;29(1):24–26. doi: 10.1038/nbt.1754.
