# Supplementary material for: Case Report: Differential Genomics and Evolution of a Meningeal Melanoma Treated With Ipilimumab and Nivolumab
Source: Front Oncol. 2022 Jan 5;11:691017. doi: 10.3389/fonc.2021.691017 (PMC8766339; doi:10.3389/fonc.2021.691017)
Supplement: Supplementary file 2 [file Table_1.docx]

***Supplementary*** [***Table***](https://www.ncbi.nlm.nih.gov/pmc/articles/PMC7565756/table/cancers-12-02362-t004/) ***1.*** Selected studies for melanoma with *GNAQ*/*GNA11* mutations.

| **Study** | **Study Type** | **N** | **Arms (n)** | **ORR (%)** | **DCR (%)** | **PFS (mo)** | **OS (mo)** |
| --- | --- | --- | --- | --- | --- | --- | --- |
| Carvajal *et al.* 2014 (1) | Phase 2 | 101 | Selumetinib (50) Chemotherapy (51) | 14 0 | NS | 3.7 1.6 | 11.8 9.1 |
| Shoushtari *et al.* 2016 (2) | Phase 2 | 39 | Trametinib (18) Trametinib + GSK2141795 ^a^ (21) | 5.5 4.8 | NS | 3.6 3.6 | NS |
| Scheulen *et al.* 2017 (3) | Phase 2 | 118 | Sorafenib | 1.7 | 66.1 | 5.5 | 14.8 |
| Patel *et al.* 2017 (4) | Phase 2 | 31 | Glembatumumab Vedotin | 6 | 61 | 3.2 | 11.8 |
| Carvajal *et al.* 2018 (5) | Phase 3 | 129 | Selumetinib + Dacarbazine (97) Placebo + Dacarbazine (32) | 3 0 | NS | 2.8 1.8 | NS |
| Sato *et al.* 2018 (6) | Phase 1/2 | 19 | Tebentafusp ^c^ | 10.5 ^d^ | - | - | NR |

**References**

1. Carvajal RD, Sosman JA, Quevedo JF, et al. Effect of selumetinib vs chemotherapy on progression-free survival in uveal melanoma: a randomized clinical trial. JAMA. 2014 Jun 18;311(23):2397-405. doi: 10.1001/jama.2014.6096.
2. Shoushtari AN, Kudchadkar RR, Panageas K, et al. A randomized phase 2 study of trametinib with or without GSK2141795 in patients with advanced uveal melanoma. J Clin Oncol. 2016;34:9511. doi: 10.1200/JCO.2016.34.15_suppl.9511.
3. Scheulen ME, Kaempgen E, Keilholz U, et al. STREAM: A randomized discontinuation, blinded, placebo-controlled phase II study of sorafenib (S) treatment of chemonaïve patients (pts) with metastatic uveal melanoma (MUM) J Clin Oncol. 2017;35:9511. doi: 10.1200/JCO.2017.35.15_suppl.9511.
4. Patel S, Lewis KD, Olencki T, et al. A phase II study of glembatumumab vedotin for metastatic uveal melanoma; Proceedings of the SMR Congress; Brisbane, Australia. 18–21 October 2017; p. 194.
5. Carvajal RD, Piperno-Neumann S, Kapiteijn E, et al. Selumetinib in Combination With Dacarbazine in Patients With Metastatic Uveal Melanoma: A Phase III, Multicenter, Randomized Trial (SUMIT) J Clin Oncol. 2018;36:1232–1239. doi: 10.1200/JCO.2017.74.1090.
6. Sato T, Nathan PD, Hernandez-Aya L, et al. Redirected T cell lysis in patients with metastatic uveal melanoma with gp100-directed TCR IMCgp100: Overall survival findings. J Clin Oncol. 2018;36:9521. doi: 10.1200/JCO.2018.36.15_suppl.9521.
